# Evaluation of Equiatomic CrMnFeCoNiCu System and Subsequent Derivation of a Non-Equiatomic MnFeCoNiCu Alloy

**DOI:** 10.3390/ma16062455

**Published:** 2023-03-19

**Authors:** Artashes Ter-Isahakyan, Thomas John Balk

**Affiliations:** Department of Chemical and Materials Engineering, University of Kentucky, 177 F. Paul Anderson Tower, Lexington, KY 40506, USA

**Keywords:** transition metal alloys and compounds, high-entropy alloys, multi-principal element alloys, solidification, microstructure

## Abstract

Investigation into non-equiatomic high-entropy alloys has grown in recent years due to questions about the role of entropy stabilization in forming single-phase solid solutions. Non-equiatomic alloys have been shown to retain the outstanding mechanical properties exhibited by their equiatomic counterparts and even improve electrical, thermal, and magnetic properties, albeit with relaxed composition bounds. However, much remains to understand the processing–structure–property relationships in all classes of so-called high-entropy alloys (HEAs). Here, we are motivated by the natural phenomena of crystal growth and equilibrium conditions to introduce a method of HEA development where controlled processing conditions determine the most probable and stable composition. This is demonstrated by cooling an equiatomic CrMnFeCoNiCu alloy from the melt steadily over 3 days (cooling rate ~4 °C/h). The result is an alloy containing large Cr-rich precipitates and an almost Cr-free matrix exhibiting compositions within the MnFeCoNiCu system (with trace amounts of Cr). From this juncture, it is argued that the most stable composition is within the CrMnFeCoNiCu system rather than the CrMnFeCoNi system. With further optimization and evaluation, a unique non-equiatomic alloy, Mn_17_Fe_21_Co_24_Ni_24_Cu_14_, is derived. The alloy solidifies and recrystallizes into a single-phase face-centered cubic (FCC) polycrystal. In addition to possible applications where Invar is currently utilized, this alloy can be used in fundamental studies that contrast its behavior with its equiatomic counterpart and shed light on the development of HEAs.

## 1. Introduction

High-entropy alloys (HEAs) are a novel class of metallic materials that have triggered a renaissance in physical metallurgy, beginning with the seminal publications on the subject in 2004 [1,2]. There are several defining characteristics for HEAs, though they are most commonly defined as an alloy containing at least five principal alloying elements, each with a concentration between 5 and 35 at.% [2]. These alloys may exhibit favorable properties compared to conventional dilute solid solutions, but their preeminent complexity and relative novelty have led to difficulties in design and exploration. Numerous studies in this field have been motivated by the primary HEA concept, which postulates that maximum configurational entropy can be achieved through equiatomic ratios, which, in turn, will stabilize single-phase solid solutions [3,4,5,6,7,8]. However, a growing number of studies have shown that entropic stabilization alone is insufficient, and the optimal balance may be found in non-equiatomic mixtures [3,6,9,10,11,12,13,14,15].

First investigated by Cantor et al. [1], the CrMnFeCoNi system, also known as the “Cantor alloy”, has served as the face-centered cubic (FCC) model alloy and the foundation for fundamental study. In this seminal work, the seemingly undiscovered multicomponent phase space with near-equiatomic compositions was investigated. Five-component, equiatomic Cr-Mn-Fe-Co-Ni alloys as well as six-component equiatomic alloys with the incorporation of Nb, Ge, Cu, Ti, or V were highlighted. The authors concluded that Cr_20_Mn_20_Fe_20_Co_20_Ni_20_ solidifies dendritically into a single-phase FCC solid solution and that the primary FCC dendritic phase is preserved with the addition of Nb, Ge, Cu, Ti, and V. Additionally, it was observed that Nb, Ti, and V can dissolve in significant amounts into the primary FCC dendritic phase, while the more electronegative Cu and Ge are rejected into the interdendritic phase. Listed in Table A1 and Table A2 are the dendritic and interdendritic compositions and lattice parameters, respectively.

In the development of the Cantor alloy, the elemental combination was assessed as optimal since nearly equiatomic ratios of CrMnFeCoNi appeared in the cast structures of equiatomic mixtures of CrMnFeCoNi—(Nb, Ge, Cu, Ti, or V). Here, we argue that the assessment of an optimal phase based on such an evaluation may be incomplete because non-equilibrium processing methods, such as induction melting, are utilized and can result in the formation of metastable or non-equilibrium phases. Given the complexities associated with multicomponent systems and the potential impacts of processing conditions, the final microstructure of a given alloy is sensitive to a vast range of parameters. Therefore, it can be argued that the observed compositions may be metastable phases that could exist in equilibrium under a given condition but not exist in equilibrium under different conditions (e.g., composition, temperature, cooling rate). True equilibrium processing would require infinitely slow cooling rates from the liquid state, which is not realistic. However, the slower the cooling rate, the closer one approaches equilibrium conditions.

Expanding on the work of Cantor et al., Yao et al. [10] and Tasan et al. [13] reported on the microstructure and mechanical properties of a non-equiatomic alloy within the Cantor system: Fe_40_Mn_27_Ni_26_Co_5_Cr_2_. These works concluded that the requirement for the equiatomic ratio can be relaxed, suggesting that the FCC phase field in the Cantor alloy system was broader than previously thought. In addition, the mechanical properties of the equiatomic and non-equiatomic alloys were found to be comparable. These results sparked further interest in non-equiatomic HEAs. Ma et al. [5,16] utilized ab initio and CALPHAD calculations to assess phase stability in Fe_x_Mn_62−x_Ni_30_Co_6_Cr_2_ alloys, investigating equilibrium phase formation at high temperatures, constituent phases after non-equilibrium solidification processes, and unfavorable segregation during solidification. Several further studies assessing the relationship between composition and phase stability in HEAs followed, particularly in the formation of a Cr-rich σ-phase.

Zaddach et al. [17] compared an NiFeCrCoMn alloy, two non-equiatomic NiFeCrCoMn alloys optimized for low stacking fault energy, and an equiatomic NiFeCrCo alloy, all produced by arc melting. Equiatomic NiFeCrCo was reported to exhibit the highest ductility and toughness after annealing, followed by Ni_18.5_Fe_18.5_Cr_18.5_Co_26_Mn_18.5_. The other non-equiatomic alloy, Ni_14_Fe_20_Cr_26_Co_20_Mn_20_, exhibited poor thermal stability, forming σ-phase intermetallics at temperatures below 1100 °C. Laplanche et al. [18] concurred, reporting that, for an overall alloy composition of Cr_26_Mn_20_Fe_20_Co_20_Ni_14_, an intermetallic σ-phase formed within the original single-phase FCC matrix after 1000 h of annealing. Laplanche et al. combined these findings with theories of precipitation kinetics and computed a TTT diagram for the equiatomic CrMnFeCoNi HEA. The σ-phase was also observed by Stepanov et al. [19] when evaluating a series of non-equiatomic ductile FCC solid solution Fe_40_Mn_28_Ni_32−x_Cr_x_ alloys. This work reported that increasing Cr content corresponded to increased yield strength and attributed this to solid solution strengthening. However, once the Cr content increased to Fe_40_Mn_28_Ni_8_Cr_24_, the alloy became brittle and contained an intermetallic σ-phase matrix. By methodically varying the amount of Co in the CrMnFeCo_x_Ni (Cantor) system, Bloomfield et al. [20] concluded that Co stabilizes the FCC solid solution relative to the σ-phase. Exposure at 700 °C and 900 °C for 1000 h was reported to result in the formation of σ-phase precipitates in the CrMnFeNi and CrMnFeCo_0.5_Ni alloys but not in the CrMnFeCo_1.5_Ni alloy.

While these studies demonstrate that the relationship between composition and phase stability in HEAs has certainly been considered, expansion beyond the circumstances around the σ-phase has been limited. The further inclusion of processing conditions—the importance of which has been emphasized earlier—to this equation beyond prolonged annealing has not been presented in the literature. Here, we propose a fabrication method applicable to both equiatomic and non-equiatomic HEAs, where controlled processing conditions determine the most probable and stable composition. This is demonstrated through a thorough experimental analysis that tests the following hypotheses:

- For HEAs, an equiatomic composition maximizes configurational entropy, but structure and phase stability are not necessarily optimized.
- CrMnFeCoNi is *not* the most stable or preferred alloy composition within the CrMnFeCoNiCu system if the processing conditions (especially a slow cooling rate from the melt) are controlled.

We approach this by investigating the effect of Cu on the Cantor alloy system—beginning by assessing the equiatomic six-component CrMnFeCoNiCu alloy and later substituting Cu for Cr in the five-component MnFeCoNiCu system. We approximate equilibrium conditions by cooling an equiatomic CrMnFeCoNiCu alloy from the melt steadily over a three-day period (~4 °C/h cooling rate). This results in the large Cr-rich precipitates found in Ref. [21] dispersed throughout a Cr-poor matrix with a composition within the MnFeCoNiCu system. From this juncture, we argue that the most stable composition is within the MnFeCoNiCu system rather than the classic CrMnFeCoNi system. Likewise, we argue that if stability is found within MnFeCoNiCu, the composition with optimal phase stability and/or mechanical properties is not equiatomic. We support this by deriving a stable, single-phase non-equiatomic Mn_17_Fe_21_Co_24_Ni_24_Cu_14_ (at.%) HEA by qualitatively evaluating the matrix composition in the slow-cooled CrMnFeCoNiCu system.

## 2. Materials and Methods

The experimental procedures for alloy processing/fabrication and testing are threefold and will be given sequentially. Experiments 1 and 2 evaluate the CrMnFeCoNiCu system: (1) initial evaluation of the CrMnFeCoNiCu system prepared by arc melting and (2) evaluation after slow cooling where a non-equiatomic Mn_17_Fe_21_Co_24_Ni_24_Cu_14_ alloy is derived. Experiment 3 involves experimental procedures regarding the newly derived non-equiatomic Mn_17_Fe_21_Co_24_Ni_24_Cu_14_ (at.%) from experiment 2.

### 2.1. CrMnFeCoNiCu Produced by Arc Melting

Initially, a 20 g ingot of CrMnFeCoNiCu was produced by arc melting pure metal pieces in a high-purity Ar atmosphere on a water-cooled copper hearth (Edmund Bühler MAM-1 Compact Arc Melter, Edmund Bühler GmbH, Bodelshausen, Germany). Mn, Fe, Co, Ni, and Cu had a purity of >99.9%, while Cr was 99.2% pure. Mn, Fe, Co, Ni, and Cr were supplied by Alfa Aesar (Ward Hill, MA, USA), and the Cu was sourced from an OFHC copper gasket (Kurt J. Lesker, Jefferson Hills, PA, USA). Ingots were flipped and remelted 4–6 times to improve compositional homogeneity. The as-cast ingots were then sectioned, and standard metallographic preparation was performed. Crystal structures of the alloy phases were analyzed with X-ray diffraction (XRD; Siemens D500, Bruker Corporation, Madison, WI, USA). Microstructure and composition were characterized in a scanning electron microscope (SEM; FEI Quanta FEG 250 and FEI Helios NanoLab 660, Thermo Fisher Scientific, Hillsboro, OR, USA) with composition measured using X-ray energy dispersive spectroscopy (EDS; FEI Helios NanoLab 660 with Oxford 80 mm^2^ EDS detector, Oxford Instruments Nanoanalysis, Concord, MA, USA). To assess the stability of composition and microstructure, homogenization and recrystallization attempts were made on an ingot slice roughly 1 mm wide. Here, the ingot was cold-rolled to a 30% thickness reduction and subsequently annealed at 1200 °C for 48 h (Lindberg/Blue M™, T_max_ = 1500 °C) in a flowing Ar environment and allowed to cool to room temperature. The sample was then prepared for metallography and characterized by XRD and SEM/EDS.

### 2.2. CrMnFeCoNiCu Produced from Slow Furnace Cooling

**Figure 1:**
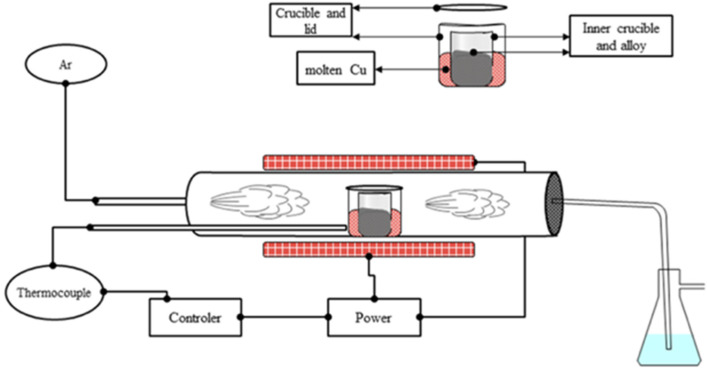
Illustration of the tube furnace setup for slow cooling. A double-crucible method was used, which incorporates the alloy in the inner crucible and a sacrificial element (Cu) in the outer crucible. Temperature is controlled by the thermocouple touching the crucible.

Motivated to control solidification and minimize thermal gradients and oxidation, the method illustrated in Figure 1 was developed. For controlled cooling, the control thermocouple was placed inside the tube furnace and in contact with the crucible. This ensures that the furnace temperature is controlled by the temperature of the crucible rather than the atmosphere of the furnace tube. A double crucible system was developed to minimize oxidation as much as possible, while also minimizing thermal gradients. The molten alloy was located in the inner crucible, which was surrounded by a sacrificial element in a larger, lidded crucible. The sacrificial element, Cu, served as an oxygen getter while also thermally homogenizing the melting crucible at high temperatures. In this setup, the alloy ingot was first prepared via arc melting and then remelted in the described setup. The thermal history consists of heating to 1475 °C at a rate of 5 °C/min followed by an equilibration dwell for 2 h, then cooling to 1200 °C at a constant rate over a period of 3 days (72 h), i.e., ~4 °C/h. Prior to heating, the furnace tube was securely capped such that no leaks were detected, and then the tube was purged with pure Ar and evacuated with a mechanical pump 3 times.

### 2.3. Preparation of Mn_17_Fe_21_Co_24_Ni_24_Cu_14_

A bulk sample of Mn_17_Fe_21_Co_24_Ni_24_Cu_14_ (at.%) weighing approximately 5 g was prepared via arc melting in a similar manner as in experiment 1. Physical and thermal homogenization was achieved by rolling a slice of an ingot to a 30% thickness reduction and subsequently annealing in a tube furnace with constant Ar flow (similar setup as shown in Figure 1). The annealing schedule was as follows: heated at 5 °C/min to 900 °C and held for 1 h, followed by heating at 10 °C/min to 1150 °C and holding for 1.5 h, then allowed to cool naturally.

### 2.4. Mechanical Testing

Samples of the equiatomic and non-equiatomic alloys produced via arc melting and homogenization were evaluated in tension using an MTS 810 Servo Hydraulic Testing System according to ASTM E8.

## 3. Results and Discussion

### 3.1. CrMnFeCoNiCu Produced by Arc Melting

**Figure 2:**
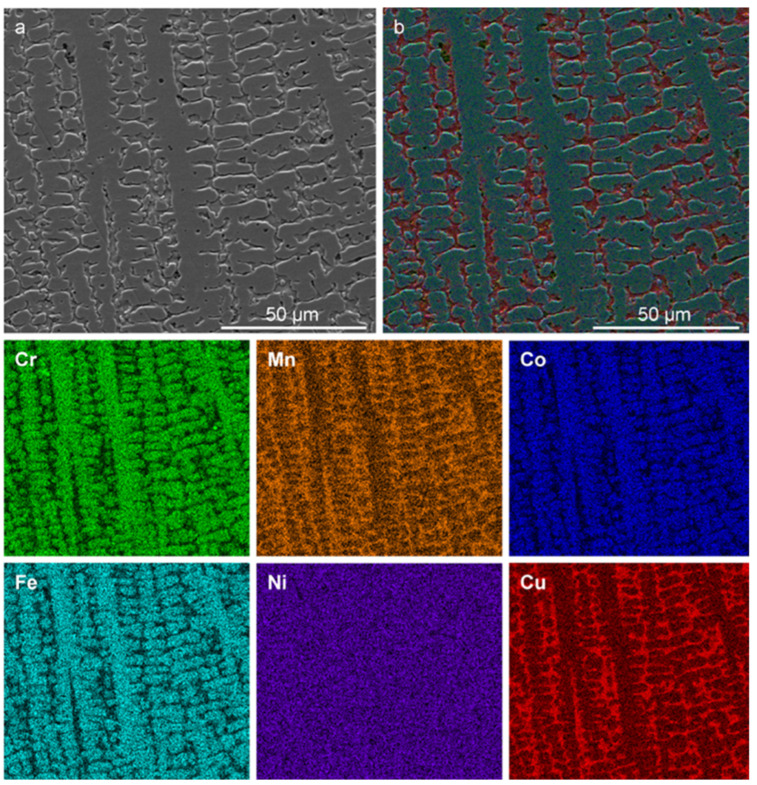
Microstructure of as-cast CrMnFeCoNiCu alloy. (**a**) SEM micrograph showing dendritic as-cast structure and (**b**) layered SEM+EDS elemental map along with elemental maps of the constituent metals.

To assess composition and microstructure, a cross-section SEM micrograph and corresponding elemental maps of the equiatomic CrMnFeCoNiCu alloy fabricated via arc melting are shown in Figure 2.

The morphology and compositional descriptions by Cantor et al. [1] are consistent with the observations and findings of the current work (see appendix A). Table 1 lists the dendritic (D) and interdendritic (ID) compositions identified by EDS. The error associated with this technique is typically ±1 at.%.

**Figure 3:**
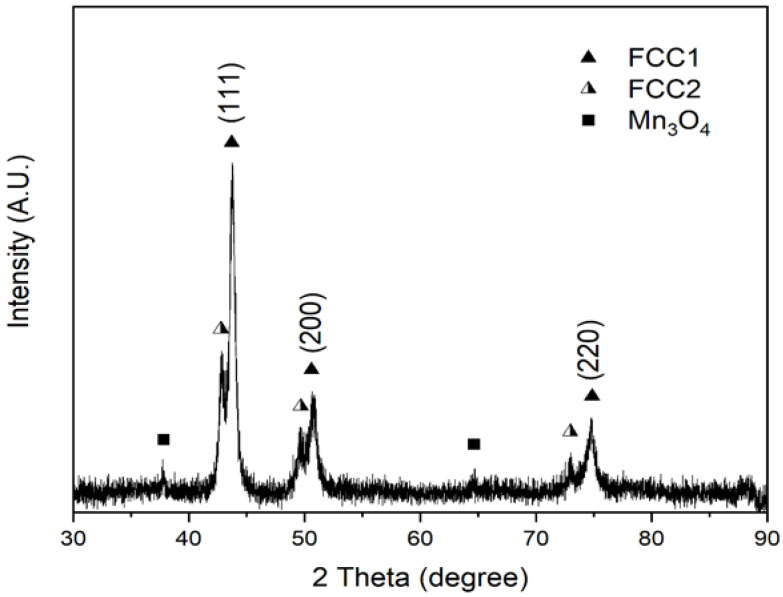
XRD pattern of equiatomic CrMnFeCoNiCu, exhibiting peaks for two FCC phases and minor amounts of MnO identified on the basis of d-spacing.

The compositional variation between D and ID further supports the claim that a Cu instability exists in FCC HEAs within the Cantor alloy family and is consistent with previous studies (Refs. [1,14]). This is hypothesized to result either from electronegativity differences or positive enthalpic contributions. The XRD diffractogram of as-cast equiatomic CrMnFeCoNiCu is shown in Figure 3, where two sets of FCC diffraction peaks are indexed along with minor amounts of MnO (Crystallography Open Database #1514241 [22,23]). The lattice parameters of the two FCC phases are listed in Table 2.

In contrast to Table A2, the lattice parameter of the FCC1 phase is correlated with the primary dendritic phase, but no corresponding information is available for the secondary FCC2 phase, which is inferred to belong to the Cu-rich ID phase.

Overall, the findings support a near equiatomic primary FCC phase with compositions near the equiatomic CrMnFeCoNi system in the as-cast state. One disadvantage of the methodology used is that arc melting, or induction melting followed by solidification, will almost certainly result in non-equilibrium microstructures. Although such processing is frequently used favorably for some industrial applications, conclusions about equilibrium microstructures based on these alloys would be speculative. This is because large amounts of undercooling and steep thermal gradients can preferentially precipitate and propagate non-equilibrium or metastable phases, and the final microstructure may be devoid of equilibrium phases.

### 3.2. CrMnFeCoNiCu after High-Temperature Heat Treatment

**Figure 4:**
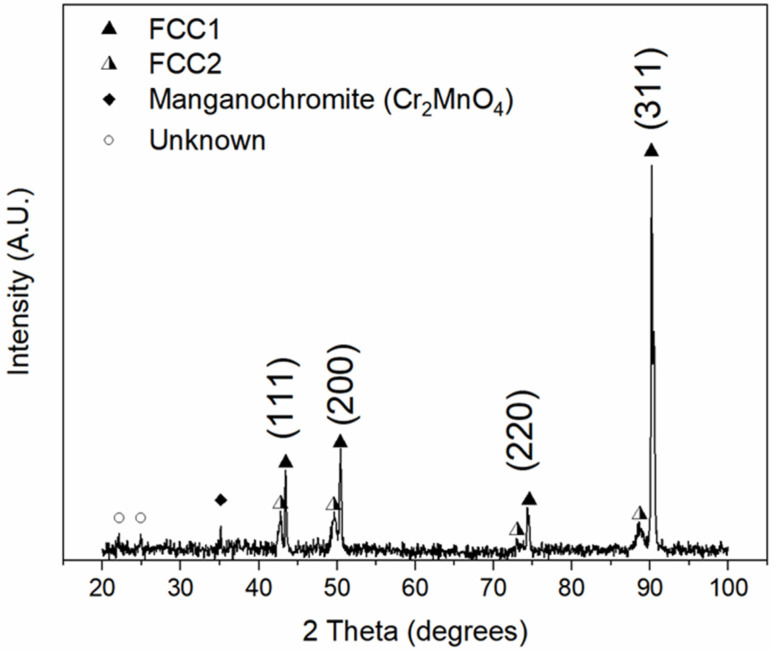
XRD pattern of equiatomic CrMnFeCoNiCu after 30% rolling reduction and annealing at 1200 °C for 48 h.

**Figure 5:**
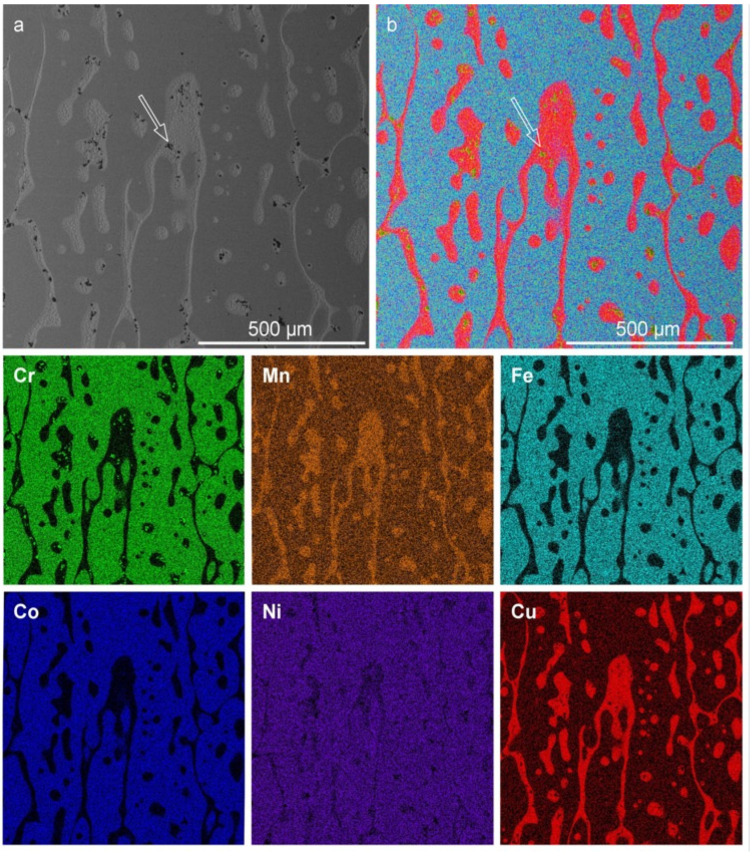
Morphology and elemental maps of the CrMnFeCoNiCu alloy after rolling and isothermal heat treatment. (**a**) Layered EDS map, (**b**) SEM micrograph, and constituent elemental maps of Cr, Mn, Fe, Co, Ni, and Cu, respectively. Arrows in (**a**,**b**) mark the presence of Cr-rich oxide inclusions.

Although the as-cast diffractogram (Figure 3) indicates two FCC phases, it is unclear whether this is a consequence of segregation or if the system intrinsically solidified into two phases. Prolonged high-temperature heat treatments usually equilibrate the microstructural morphology and homogenize the chemical compositions of each constituent phase. As such, a slice of the as-cast ingot was rolled to 30% reduction and isothermally annealed at 1200 °C for 48 h, resulting in the diffractogram in Figure 4. A similar dual-phase FCC diffraction pattern is evident, as well as three additional peaks, one of which is manganochromite (Crystallography Open Database #9012051 [23,24]). In addition, the FCC1 peaks partitioned and narrowed while also exhibiting a (311)-oriented texture. The measured lattice parameters for the FCC1 and FCC2 phases are 3.603 and 3.659 Å, respectively. In contrast to the values in Table 2, the lattice parameter remained essentially unchanged for the FCC2 phase, while a slight increase was observed for the FCC1 phase. This disparity is hypothesized to originate from varied amounts of Cu dissolved in the FCC1 phases and/or to the magnitude of residual stresses present in the as-cast vs. annealed states. The corresponding microstructure is shown in Figure 5.

Compared to the as-cast microstructure, the morphology evolved significantly while maintaining the duplex aspect and similar elemental partitioning. The microstructure is distinct with coalesced and elongated Cu-depleted phases, surrounded by Cu-rich channels or encapsulating Cu-rich nodules. Interestingly, the morphology resembles a “lava-lamp,” which implies immiscibility between the two FCC phases. The presence of Cr-rich particles was observed within Cu-rich channels (marked with arrows in Figure 5a,b). EDS analysis revealed that the particles consist mostly of Cr, Mn, and O, which corresponds with the manganochromite observed in XRD. The average measured compositions of these two phases are summarized in Table 3.

**Figure 6:**
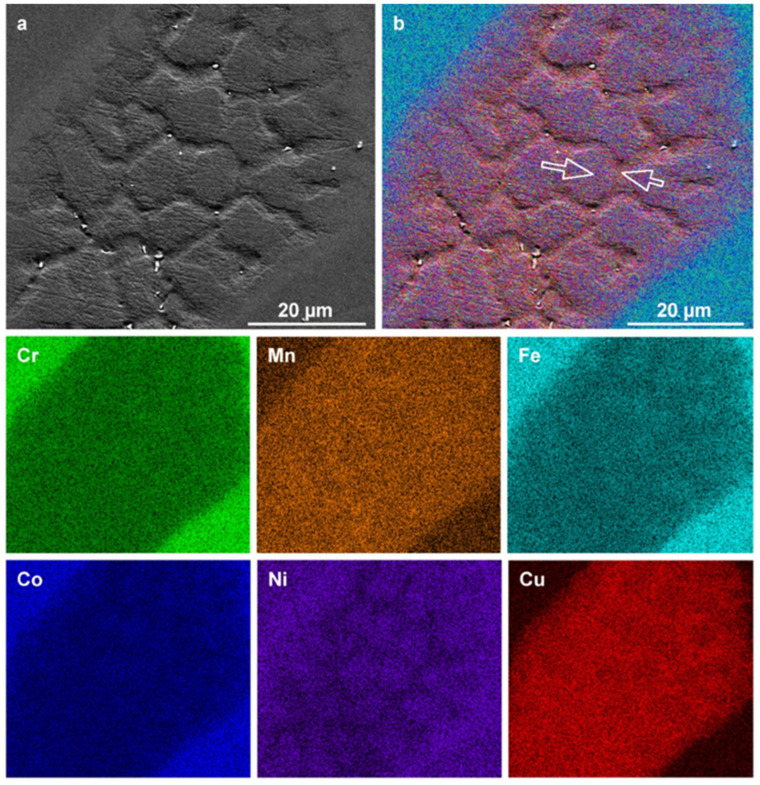
Morphology and elemental maps of a heat-treated CrMnFeCoNiCu alloy, focusing on a Cu-rich zone. (**a**) SEM micrograph and (**b**) corresponding layered EDS map and constituent elemental maps of Cr, Mn, Fe, Co, Ni, and Cu, respectively. Arrows indicate a region exhibiting compositional and morphological variations.

Compared to the composition from the as-cast state in Table 1, there are no significant differences in composition for the Cu-depleted phase compared to the D phase previously defined. There is a slight decrease in Cr content, which may be due to measured differences from regional variations in composition or may be due to depletion caused by the formation of oxides. Conversely, the Cu-rich phase differs from the ID phase as demonstrated by a significant reduction of Cr, Fe, and Co. However, the composition is not homogeneous within the bulk of the phase. This can be observed at higher magnification as shown in Figure 4, Figure 5 and Figure 6.

The spheroidal shape in combination with compositional fluctuations mimic a cored microstructure. The formation of a cored structure implies that the Cu-rich regions transformed to a liquid state during the heat treatment process, and subsequent cooling and solidification resulted in coring. This also implies that the alloy was in a state of simultaneous solid and liquid. Given that the primary Cu-depleted phase remained compositionally constant while evolving morphologically, a monotectic reaction is expected. This is commonly observed in alloys containing insoluble elements, which is the case for Cu-Cr, Cu-Co, and Cu-Fe alloys [25].

The preceding evaluation demonstrates that there is little to no tendency towards compositional homogenization after high-temperature heat treatments. The associated high-temperature microstructure is observed to have a distinct Cu-rich phase and a Cu-depleted primary phase. The formation of a “lava-lamp” morphology inside the microstructure is a unique and intriguing feature and may be a consequence of interfacial instability within the cast structure. The development of such morphology suggests that the specific alloy system has a high propensity for dynamic microstructural evolution.

### 3.3. Slow Cooling of Equiatomic CrMnFeCoNiCu from the Liquid State

The rapid cooling rates and steep thermal gradients associated with arc melting or induction melting are bound to lead to non-equilibrium solidification, which explains the microstructures observed above and the potential for significant microstructural evolution after high-temperature annealing. As a result, we conclude that the possible phase separations are currently unknown and unexplored. Equilibrium solidification, which results in the formation of equilibrium microstructures, can only occur when solidification occurs over geological time scales. Consequently, in practice, solidification cannot occur at equilibrium. However, it is logical that degrees of deviation from equilibrium occur and form a hierarchy that corresponds to increasing cooling rates [10]. It is important to note here that some equilibrium phases, particularly those with complicated crystal structures, have slow nucleation and/or interface attachment kinetics. As a result, they may be absent in microstructures produced by fast cooling rates or even after prolonged annealing [26].

**Figure 7:**
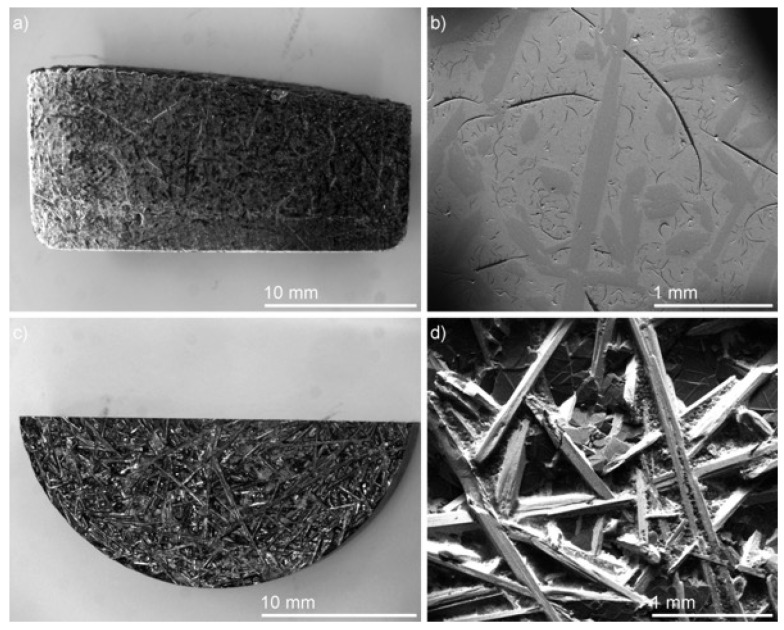
Section views of the as-cast ingot. (**a**) Cross-section optical micrograph and (**b**) corresponding SEM micrograph showing phase morphology. (**c**) Plan-view image of as-cast ingot showing acicular precipitates on the surface and (**d**) corresponding SEM micrograph.

**Figure 8:**
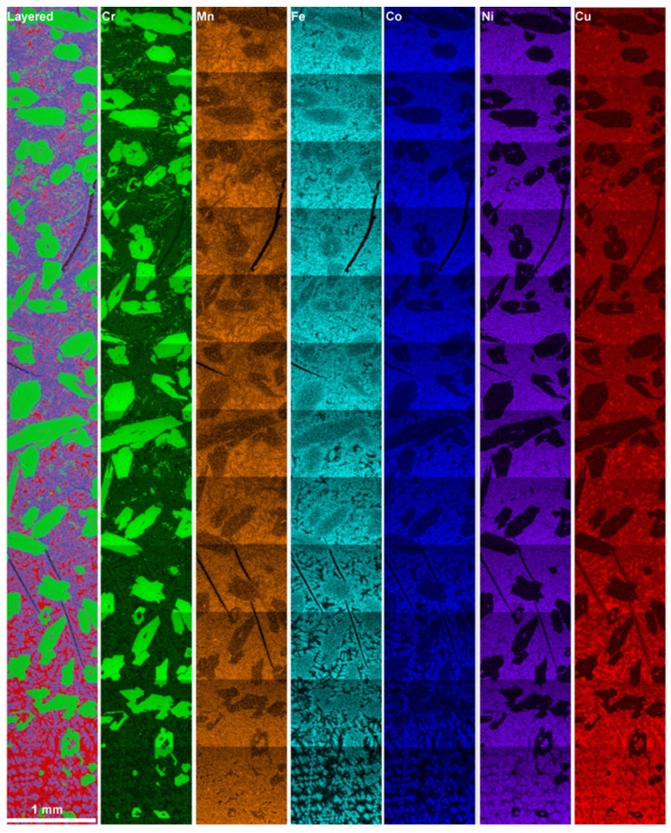
Image-stitched layered EDS elemental map of a 1 mm wide cross-section from bottom to top, with constituent elemental maps of Cr, Mn, Fe, Co, Ni, and Cu, respectively.

Figure 7 shows a cross-section of the ingot resulting from slow cooling of equiatomic CrMnFeCoNiCu from the liquid state and reveals significant morphological changes versus the observations presented earlier. The top of the ingot consists of accumulated macro-crystallites forming a distinct acicular morphology. The side cross-section reveals a composite of large macro-precipitates within a matrix with a high density of micro- and macro-cracks. EDS elemental analysis of a 1 mm wide section spanning from the top to the bottom of the ingot is presented in Figure 8.

The map spectrum is summarized in Table 4 and indicates only minor deviations from the nominal equiatomic composition, except for Mn. Evaporative loss of Mn likely occurred, given its characteristic tendency to evaporate at elevated temperatures. However, given that measurement error margins are typically 1–2 at.%, a roughly constant composition can be assumed throughout the process cycle.

**Figure 9:**
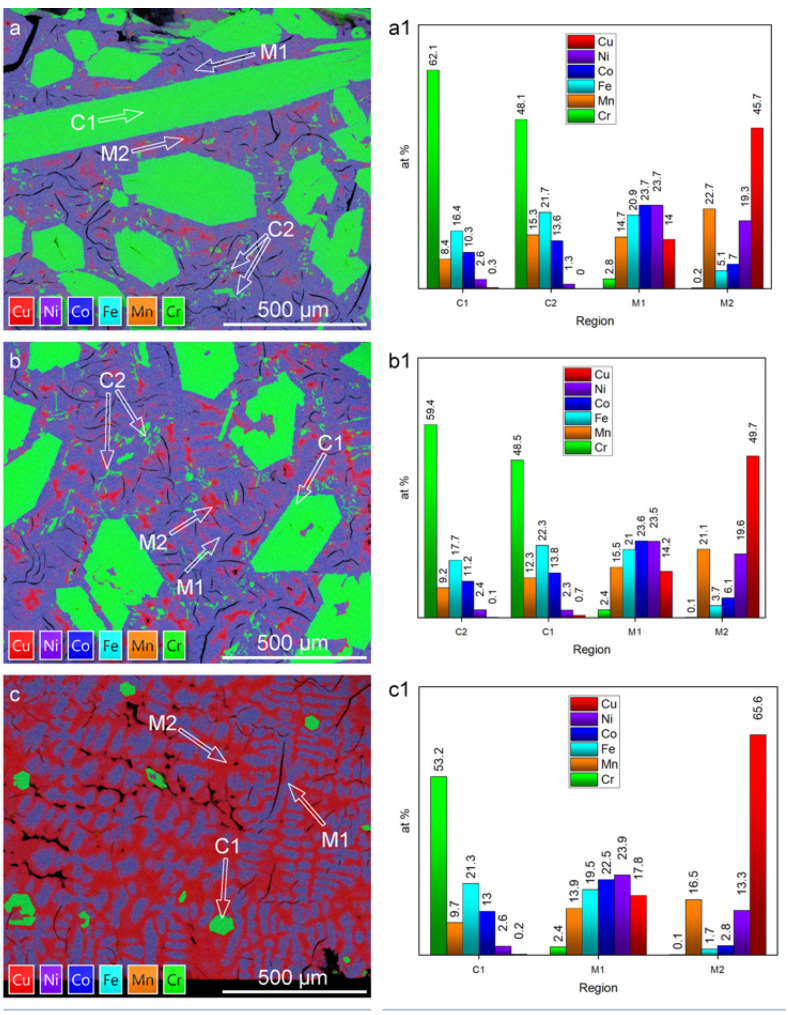
Layered EDS maps taken from regions of interest from the (**a**) top, (**b**) middle, and (**c**) bottom of the ingot. Bar charts quantifying the composition of microstructural features C1, C2, M1, and M2 are shown for regions of interest at the (**a1**) top, (**b1**) middle, and (**c1**) bottom of the sample.

In comparison to microstructures formed by arc melting or annealing, the slow-cooled microstructure is completely distinct. This is evidenced by the absence of previously observed, compositionally dissimilar segregations. The slow-cooled microstructure is defined by two main characteristics: Cr-rich crystallites and a Cu-segregated matrix. The topological distribution of crystallites varies from top to bottom, where the frequency is lowest at the bottom and highest at the top of the ingot. The matrix exhibits a segregated morphology with Cu-enriched pockets accumulating at the bottom and decreasing toward the top of the ingot. Detailed analysis can be summarized by evaluating regions of interest from the top, middle, and bottom, as shown in Figure 9.

Overall, four (C1, C2, M1 and M2) compositionally and morphologically distinct features can be identified and are representative of the microstructure of the cast ingot. The size, shape, composition, and distribution of these features vary across the microstructure. Overall, regional differences in composition and morphology are significant only when compared to compositions analyzed from the bottom of the cast ingot. However, useful insights about the solubility or segregation tendencies of the alloying components can be drawn.

The crystallites and coagulates are characterized within the compositional bounds of Cr_53-62_Mn_8-10_Fe_16-21_Co_10-13_Ni_2.5_Cu_~0_ and Cr_48-49_Mn_12-15_Fe_21-22_Co_14_Ni_1-2_Cu_~0,_ respectively. Variation in trace amounts of Ni and Cu imply that the crystallites are chemically defined by Cr, Mn, Fe, and Co. The upper and lower bounds follow a trend, in which the upper bounds of Cr are balanced by the lower bounds of Mn, Fe, and Co, and vice versa. The variation is likely due to proportional dissolution of Mn/Fe/Co, which then depends on the local chemical environment that surrounds the crystallites. Moreover, bounds describing the primary dendritic and interdendritic components of the matrix are Cr_2-3_Mn_14-16_Fe_19-21_Co_22-24_Ni_23-24_Cu_14-18_ and Cr_~0_Mn_16.5-23_Fe_2-5_Co_3-7_Ni_13-19_Cu_46-66_, respectively. The rejection of Ni and Cu from the crystallites contrasts the rejection of Cr from the matrix, suggesting that the matrix is defined primarily by Mn, Fe, Co, Ni, and Cu.

The microstructural variations can be explained by considering buoyant and gravitational factors. The sparse distribution of crystallites near the bottom and gradual accumulation towards the top implies that they form first, with a lower density than the surrounding liquid. As a result of buoyant forces, crystallites float towards the top and cover the surface. The morphological gradient of the matrix can be explained similarly. If dendritic solidification is assumed, the formation of the matrix dendrites is accompanied by the rejection of lower melting point solutes into the liquid. In this context, the solutes can be ascribed to Cu/Mn that saturate and form a Cu-rich liquid. Given that Cu is the heaviest element, it would be expected that gravitational forces will lead to accumulation towards the bottom of cast samples.

**Figure 10:**
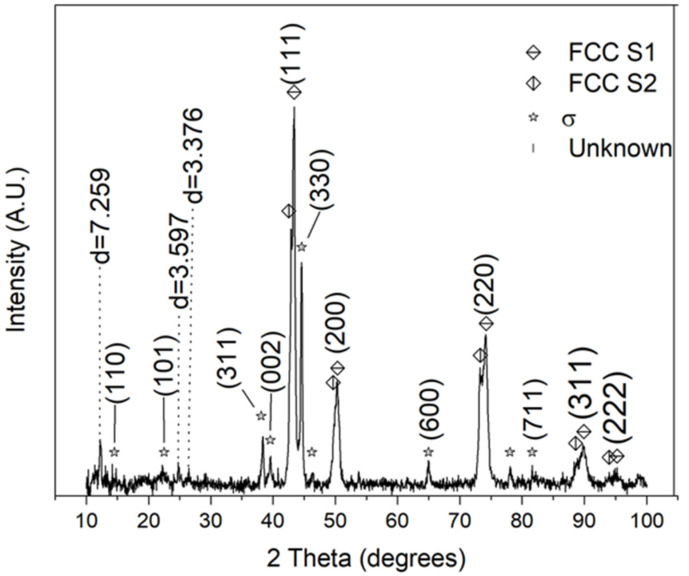
XRD pattern of CrMnFeCoNiCu alloy after slow cooling and exhibiting multiple phases. For unindexed peaks, the interplanar spacing *d* is given in angstroms.

Significant structural changes are also evident, as shown by the XRD diffraction scan in Figure 10. In contrast to the diffractograms shown in Figure 2 and Figure 3, the FCC diffraction peaks are more similar to each other (lower amount of peak splitting), along with notable differences including the appearance of a σ-phase and numerous unidentified peaks. For the FCC phases, the calculated lattice constants are 3.613 Å (FCC-S1) and 3.649 Å (FCC-S2), respectively. The FCC phases likely belong to the matrix compositions, where the Cu-rich matrix results in a larger lattice constant. The σ-phase likely corresponds to the Cr-rich crystallites since Cr is a well-known stabilizer of the σ-phase [27,28], as discussed previously. The lattice parameters for the σ-phase were found to be *a* = *b* = 8.614 Å and *c* = 4.569 Å, indicating a slightly lower volume than what was reported in Ref. [8]. For the unindexed diffraction peaks, the COD database available (Ref. [23]) failed to match any compound or phase that contains elements present in the alloy. The peak with *d* = 3.597 Å is somewhat consistent with the lattice parameters of the FCC phase and may represent an ordered FCC phase or may be an ordered tetragonal L1_0_ phase. The peak with *d* = 7.259 Å is by far the most unusual. Firstly, the value is roughly double that of the lattice parameter of the FCC-S1 phase, suggesting a possible structural relationship. Secondly, the presence of a peak at low angles suggests a very large lattice structure. Possible explanations may include the transformation of martensite, or the peak may result from an interface between the σ-phase and the FCC phase.

### 3.4. Derivation of MnFeCoNiCu from the Slow-Cooled Microstructure

Configurational entropy is widely acknowledged as the fundamental quantity that explains the formation of solid solutions. Within the scope of HEAs, configurational entropy is maximized by utilizing the multi-principal element concept, which in turn is thought to facilitate solid solution formation by lowering the free energy according to $∆G_{mix}=-T∆S_{conf}$. This assumption is valid for ideal solutions, $∆H_{mix}=0$, or when $∆H_{mix}\neq 0$, granted that deviations from ideal behavior are small. The equiatomic CrMnFeCoNiCu system solidifies into two compositionally distinct phases when prepared by arc melting and into at least three compositionally distinct phases when cooled slowly from the melt. Decomposition highlights the notion of non-ideality, more specifically, the influence of enthalpy and the interactions between constituent elements. In the first case, a primary FCC structure is compositionally defined by a nearly equiatomic CrMnFeCoNi system and the rejection of Cu. Such a tendency can be interpreted by evaluating the binary enthalpies of the constituents given in Table 5.

Aside from the Cr-Mn pair, all binary pairs within the Cr-Mn-Fe-Co-Ni system are zero or negative. This contrasts the positive values for binary pairs between Cu and all other elements within the system. Accordingly, this would lead to an expected de-mixing of Cu based on a positive enthalpic contribution. Moreover, the enthalpy of mixing for a system containing Cr-Mn-Fe-Co-Ni will always be a net negative near equiatomic ratios, and on the order of −4 kJ/mol for an equiatomic alloy. This viewpoint most likely explains the formation of Cr-rich crystallites.

The formation of Cr-rich crystallites emphasizes the hypothesis that a thermodynamically favorable solid solution is not a Cr-containing, near-equiatomic phase within the CrMnFeCoNiCu system, and any such forms are in a quenched disordered state and will decompose if kinetically favorable conditions are met. Furthermore, the composition of a primary matrix phase formed after slow cooling is nearly equiatomic and composed primarily of Mn-Fe-Co-Ni-Cu with trace amounts of Cr—within the compositional bounds of Cr_2-3_Mn_14-16_Fe_19-21_Co_22-24_Ni_23-24_Cu_14-18_. In this respect, the near equiatomic bounds imply a likelihood of forming a solid solution state. This is especially noteworthy given that the precursor for the Cantor alloy is often mentioned as a compositionally favorable primary phase within systems containing Cr-Mn-Fe-Co-Ni. Table 6 provides an overview of compositions derived from elemental analysis of primary dendritic regions from the bottom, middle, and top zones, along with calculated values of mixing enthalpy and entropy.

The calculated thermodynamic values show that the mixing entropy remains constant regardless of the region. On the other hand, the enthalpy of mixing for the composition observed in the bottom zone is found to be two times higher than that observed in the middle and top zones. This is quite interesting considering the narrow compositional bounds, as it demonstrates how minor compositional fluctuations may lead to significant enthalpic contributions without impacting the entropic term. The difference, in the case of the bottom zone, is the consequence of a higher Cu content with a positive contribution, coupled with a lower Mn content with a negative contribution.

As was previously discussed, a solid solution state with random atomic configurations is enabled by a high mixing entropy and a near-zero mixing enthalpy. This in turn facilitates ideal or nearly ideal solution behavior. Accordingly, the compositions in the top and middle zones contain the most effective elemental ratios as potential solid solutions. The ratios can be further optimized by excluding Cr and adding Mn in its place. This can be argued by considering several points: (i) Cr positively interacts with Mn as well as Cu and contributes to a higher mixing enthalpy; (ii) a minor increase in Mn lowers the overall mixing enthalpy; (iii) the processing of the melt resulted in an evaporative loss of Mn on the order of 2 at.%; and (iv) the addition of Mn would compensate this potential loss. Accordingly, the optimized composition equates to Mn_17_Fe_21_Co_24_Ni_24_Cu_14_, which lowers the mixing enthalpy to 0.49 kJ/mol and results in a mixing entropy of 1.59R.

**Figure 11:**
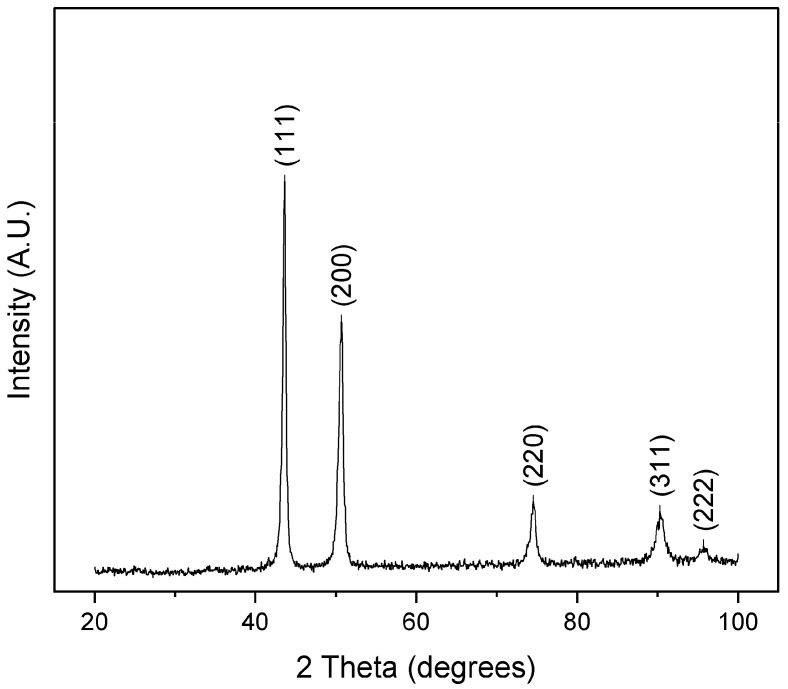
XRD diffractogram of as-cast Mn_17_Fe_21_Co_24_Ni_24_Cu_14_ alloy showing single phase FCC diffraction peaks.

**Figure 12:**
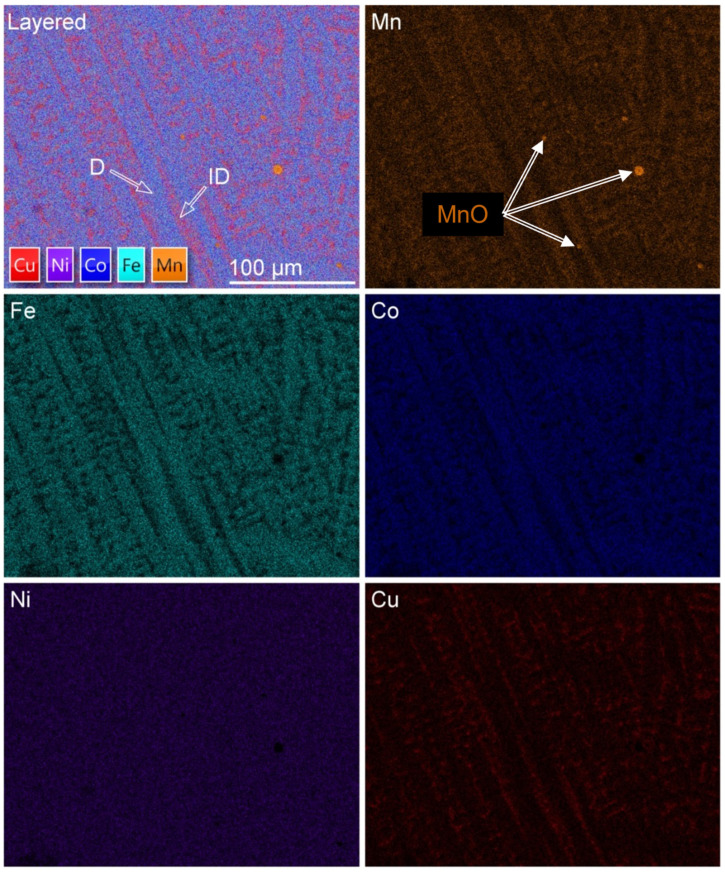
Layered EDS map and constituent elemental maps from an arc-melted, Mn_17_Fe_21_Co_24_Ni_24_Cu_14_ alloy cross-section.

To determine whether the derived composition is viable, a 5 g button was arc-melted for evaluation. XRD structural analysis of a cross-section results in the diffraction patterns shown in Figure 11. The alloy solidifies into an FCC structure with a calculated lattice parameter *a* = 3.594 Å. Microstructural and elemental analyses of a region in the same cross-section are shown in Figure 12.

The solidified microstructure is dendritic, with Fe/Co and Cu/Mn-enriched dendritic and interdendritic regions, respectively. The composition of each region is presented in the Table 7.

As expected, elements with lower melting temperatures are ejected into the interdendritic region during dendritic solidification. However, in interdendritic regions, the Ni content, which has the highest melting point, is nearly identical or marginally higher. This is quite interesting and suggests that Ni’s interaction within the system is neutral.

**Figure 13:**
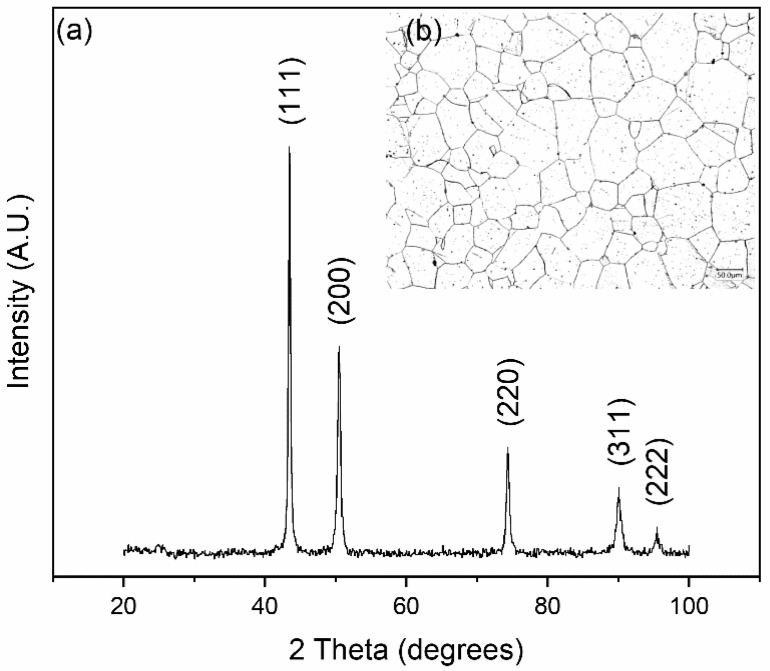
(**a**) XRD diffractogram of homogenized Mn_17_Fe_21_Co_24_Ni_24_Cu_14_ alloy showing single-phase FCC diffraction peaks, and (**b**) optical micrograph showing a nearly equiaxed grain structure.

**Figure 14:**
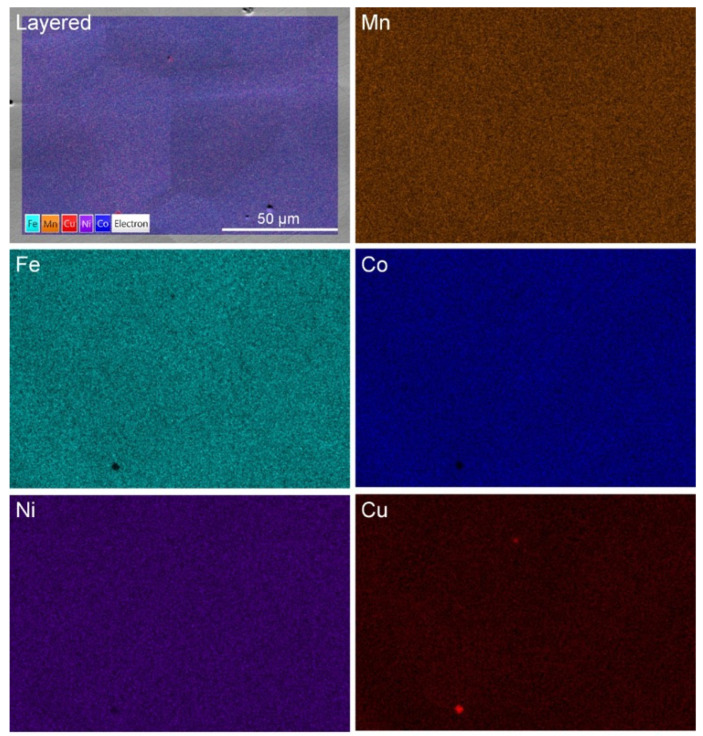
Layered EDS map and constituent elemental maps of homogenized Mn_17_Fe_21_Co_24_Ni_24_Cu_14_ alloy. A small number of Cu-enriched oxide inclusions are observed.

Further homogenization of the alloy results in the XRD pattern shown in Figure 13. The alloy recrystallizes into a microstructure that is nearly equiaxed and exhibits FCC diffraction peaks. The calculated lattice parameter is *a* = 3.605 Å, which is slightly greater than the lattice parameter in the as-cast sample (*a* = 3.594 Å). However, even slight variations are indicative of large differences when lattice parameters are compared. The variation may be a result of casting-related residual stresses, or it may be a result of the homogenization process. The latter case is more likely, as the primary dendritic phase, enriched in Co/Fe, would result in a lower lattice parameter. Elemental maps of the homogenized microstructure are shown in Figure 14.

EDS analysis of the homogenized microstructure reveals no preferential elemental clustering. It should be noted, however, that the maps represent analysis within the spatial resolution limits of the EDS technique, which is ~1 μm, and clustering may occur at the nanoscale but not be observable at higher length scales using EDS in the SEM.

### 3.5. Tensile Properties of Homogenized Alloys

**Figure 15:**
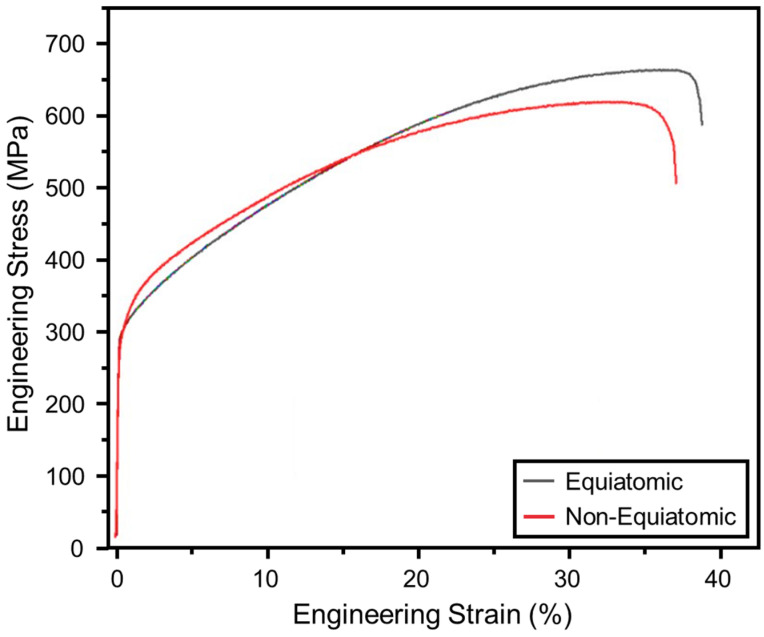
Tensile stress–strain curves for both the equiatomic and non-equiatomic alloys.

Tensile stress–strain curves of the equiatomic and non-equiatomic alloys produced via furnace melting (and subsequently homogenized) are compared in Figure 15. The individual curves are difficult to distinguish in the elastic loading regime, suggesting comparable stiffness values. The difference between the two elastic moduli values was found to be within <5%—178 GPa for the equiatomic alloy and 184 GPa for the non-equiatomic alloy. The yield strengths were also similar—313 MPa for the equiatomic alloy and 334 MPa for the non-equiatomic alloy. This behavior of the equiatomic alloy aligns with results reported elsewhere [19]. The values of work hardening rate and ultimate tensile strength (UTS) deviated more noticeably. In contrast to the yield strengths, the equiatomic alloy has a higher tensile strength than the non-equiatomic alloy. The UTS for the equiatomic alloy (~650 MPa) is consistent with the findings of another study [30]. Though further investigation beyond the scope of the current work would be required to fully elucidate the relationships between processing, structure, and mechanical properties, generally, the comparable mechanical behavior of the two alloys reported here is strong evidence that desirable properties of equiatomic alloys can also be achieved with non-equiatomic alloys, albeit without the severe constraints on composition—a sentiment also echoed by other researchers [10,12,13].

## 4. Summary and Conclusions

In this work, the association between thermal history and resultant phases and microstructures are investigated for the equiatomic CrMnFeCoNiCu system. By carefully controlling the thermal processing history of the system, the alloy was extensively evaluated and the following summarizes the key results:

- When prepared by arc melting and casting, this alloy system decomposes into a primary FCC phase resembling the Cantor alloy and a secondary FCC solid solution phase enriched with Cu. The primary and secondary FCC phases remain compositionally partitioned after high-temperature annealing, with no tendency towards homogenization. However, significant morphological evolution takes place resulting in the formation of a “lava-lamp” morphology that is characteristic of immiscible alloys. To this effect, it is postulated that the system forms a duplex microstructure owing to a monotectic reaction.
- The work further introduces a method applicable to HEA development, wherein controlled processing conditions dictate the most probable and stable composition. The method is motivated by the fact that non-equilibrium methods may generate non-equilibrium alloys and compositional partitioning. The method is utilized to cool an ingot of CrMnFeCoNiCu from the melt, slowly and steadily over a period of 3 days. The resulting microstructure is significantly different, with Cr transitioning from a solid solution component to a Cr-rich intermetallic. Furthermore, the matrix of the microstructure forms a primary dendritic phase with compositions within the MnFeCoNiCu system.
- Through evaluation and optimization of the primary dendritic phase, a unique non-equiatomic alloy, Mn_17_Fe_21_Co_24_Ni_24_Cu_14_, is derived. The combination and concentration of the constituents are distinctive and result in a mixing enthalpy of 0.49 kJ/mol, which resembles a regular solution.
- The alloy solidifies and recrystallizes into a single-phase FCC polycrystal. This unique non-equiatomic alloy can be utilized for fundamental studies by evaluating the equiatomic counterpart and emphasizing concepts related to composition-microstructure-property relations. It has the advantage of excluding σ-phase forming constituents.
- Together, the results emphasize the importance of enthalpic contributions within the scope of HEAs. Firstly, they demonstrate that even at high temperatures, a large mixing entropy does not always facilitate mixing of the constituent elements. This is evidenced by the duplex structure that remains partitioned after high-temperature annealing. Furthermore, the results demonstrate how the variation in cooling rates (from the melt) may significantly change the observed microstructure and phases. This strongly suggests that a well-defined, controlled thermal history is crucial in evaluating the microstructure of HEAs and further experiments utilizing this method may potentially elucidate outstanding issues in HEA development.

## Figures and Tables

**Table 1:** Dendritic (D) and interdendritic (ID) compositions (at.%).

Region	Cr	Mn	Fe	Co	Ni	Cu
D	20.13	13.75	21.00	20.78	16.85	7.50
ID	3.52	22.52	2.57	4.43	15.00	51.97

**Table 2:** Calculated lattice constants of FCC1 and FCC2 phases.

Phase	Lattice Parameter (Å)
FCC1	3.588
FCC2	3.658

**Table 3:** Compositions of the Cu-rich and Cu-depleted regions after high-temperature heat treatments.

Region	Cr	Mn	Fe	Co	Ni	Cu
Cu-rich	1.1	25.4	1	1.6	10.9	60.1
Cu-depleted	19.7	12.1	21.5	21.5	17.8	7.3

**Table 4:** Nominal (planned) versus actual (measured) compositions of the cast ingot.

Element	Planned at.%	Measured at.%
Cr	16.7	17.2
Mn	16.7	14.8
Fe	16.7	16.9
Co	16.7	17.2
Ni	16.7	17.2
Cu	16.7	16.9

**Table 5:** Values of mixing enthalpy calculated by Miedem’s model of binary atomic pairs between all constituent elements. Taken from Ref. [29].

$∆H_{mix}\;(\frac{kJ}{mol})$	Cr	Mn	Fe	Co	Ni	Cu
Cr	0	2	−1	−4	−7	12
Mn	-	0	0	−5	−8	4
Fe	-	-	0	−1	−2	13
Co	-	-	-	0	0	6
Ni	-	-	-	-	0	4
Cu	-	-	-	-	-	0

**Table 6:** Review of regional compositions (at.%) taken from the bottom, middle, and top zones of samples; respective thermodynamic parameters are also listed.

Region	Cr	Mn	Fe	Co	Ni	Cu	$\frac{-∆S_{mix}}{R}$	$∆H_{mix}\;\left(\mathrm{kJ}/\mathrm{mol}\right)$
Bottom	2.4	13.9	19.5	22.5	23.9	17.8	1.7	1.52
Middle	2.4	15.5	21.0	23.6	23.5	14.2	1.7	0.68
Top	2.8	14.7	20.9	23.7	23.7	14.0	1.7	0.68

**Table 7:** Compositions of dendritic (D) and interdendritic (ID) regions measured by EDS.

Region/Element	Mn	Fe	Co	Ni	Cu
D	11.4	27.3	29.5	23.1	8.7
ID	20.2	15.9	18.0	24.8	21.2

## Data Availability

The data presented in this study are available on request from the corresponding author.

## Appendix A

### Overview of the CrMnFeCoNi-(Nb, Ge, Cu, Ti, or V) Systems

The dendritic and interdendritic compositions and lattice parameters of the classic Cantor alloys, reported in Ref. [1] and discussed in the Introduction, are listed below in Table A1 and Table A2, respectively.

**Table A1:** Dendritic (D) and interdendritic (ID) compositions (at.%), as listed in Ref. [1].

Alloy	Region	Fe	Cr	Mn	Ni	Co	Nb	Ge	Cu	Ti	V
CrMnFeCoNi	D	20.2	20.5	19.4	19.5	20.4					
	ID	16.1	27.0	23.0	17.0	17.0					
Nb	D	16.5	15.7	12.0	13.0	17.8	25.0				
	ID	15.5	19.4	25.4	23.7	14.2	1.9				
Ge	D	21.7	21.0	12.0	13.5	19.2		12.6			
	ID	8.9	11.0	20.3	22.3	13.5		24.0			
Cu	D	21.6	18.4	17.7	16.9	23.7			6.8		
	ID	3.3	3.6	24.4	15.6	5.9			47.4		
Ti	D	19.7	7.3	14.3	13.5	20.5				24.8	
	ID	18.4	10.1	24.0	22.7	15.9				8.9	
V	D	18.8	19.5	7.7	25.6	18.3					18.9
	ID	18.4	16.4	8.0	21.5	19.6					16.2

**Table A2:** Lattice parameters (in Å) from XRD measurements of FCC and BCC phases, based on results report in [1].

Alloy	FCC	BCC
CrMnFeCoNi	3.59	
Nb	3.62	
Ge	3.58	2.29
Cu	3.59	
Ti	3.64	4.82
V	3.58	
